# The phylogenetic signal of species co-occurrence in high-diversity shrublands: different patterns for fire-killed and fire-resistant species

**DOI:** 10.1186/1472-6785-12-21

**Published:** 2012-09-27

**Authors:** Marcel Cardillo

**Affiliations:** 1 Centre for Macroevolution and Macroecology, Research School of Biology, Australian National University, Canberra, A.C.T. 0200, Australia

**Keywords:** Coexistence, Competition, Co-occurrence matrix, Phylogenetic community ecology, Phylogenetic conservatism, Regeneration strategy, Southwestern Australia

## Abstract

**Background:**

Using phylogenies in community ecology is now commonplace, but typically, studies assume and test for a single common phylogenetic signal for all species in a community, at a given scale. A possibility that remains little-explored is that species differing in demographic or ecological attributes, or facing different selective pressures, show different community phylogenetic patterns, even within the same communities. Here I compare community phylogenetic patterns for fire-killed and fire-resistant *Banksia* species in the fire-prone shrublands of southwest Australia.

**Results:**

Using new Bayesian phylogenies of *Banksia,* together with ecological trait data and abundance data from 24 field sites, I find that fire regeneration mode influences the phylogenetic and phenotypic signal of species co-occurrence patterns. Fire-killed species (reseeders) show patterns of phylogenetic and phenotypic repulsion consistent with competition-driven niche differentiation, but there are no such patterns for fire-resistant species (resprouters). For pairs of species that differ in fire response, co-occurrence is mediated by environmental filtering based on similarity in edaphic preferences.

**Conclusions:**

These results suggest that it may be simplistic to characterize an entire community by a single structuring process, such as competition or environmental filtering. For this reason, community analyses based on pairwise species co-occurrence patterns may be more informative than those based on whole-community structure metrics.

## Background

Phylogenetic information is now commonly employed in analyses of species co-occurrence and community assembly, as a way of explaining patterns of species distributions and diversity [1-4]. Community phylogenetic studies have increased steadily in sophistication over the past decade. For example, the effects of spatial and phylogenetic scale have been explored [5-7], and null models for phylogenetic community structure have been developed [6,8]. More generally, the idea that patterns of phylogenetic attraction (where close relatives are more likely to co-occur than expected by chance) and repulsion (where close relatives are less likely to co-occur) can act as a proxy for ecological processes is now regarded more critically. It is generally agreed that phylogenetic and phenotypic patterns should be analysed simultaneously if informative inferences about processes are to made [6].

One assumption that remains common in community phylogenetics, however, is the idea that the structure of a community is governed by an overarching process, such as competition or environmental filtering, that applies to all species in the community. This assumption is implicit in whole-community metrics of phylogenetic community structure such as NRI and NTI [4]. While some studies provide evidence for the simultaneous operation of competition and environmental filtering, often depending on spatial or phylogenetic scale [5-7], the assumption that the same process applies to all species in a community, at a given scale, is typically still made. However, ecological processes such as competitive exclusion occur at the level of individuals, populations or species, not communities. The structure of a community is simply an emergent property that results from the influence of such processes on the combined distributions of multiple species within a region. Therefore, it is possible that whole-community metrics obscure much informative variation in phylogenetic structure by “averaging out” the patterns of occurrence or abundance of different species within a community. An alternative approach is to analyse the phylogenetic and phenotypic signal of pairwise co-occurrences among species across a set of communities (e.g. [9-11]. This approach treats species pairs, rather than communities, as the units of analysis, more easily allowing patterns of co-occurrence to be examined separately for species with different demographic and ecological attributes.

In this study I compare the phylogenetic and phenotypic signal of co-occurrence patterns of plant species that differ in fire-regeneration strategy, in the Mediterranean-climate shrublands of southwestern Australia. This ecosystem type is exceptionally species-rich, but is still poorly represented among community phylogenetic studies (but see [11-13]. In these shrublands, fires are frequent, with average recurrence intervals around 10–15 years [14]. Some authors have argued that in these highly disturbed, non-equilibrium communities, classic theories of species coexistence through niche differentiation are less realistic than models of coexistence based on lottery recruitment and the availability of transient niches [14-19]. Other studies in Mediterranean shrublands have found evidence for patterns that may be competition-driven, such as niche differentiation [20] or phylogenetic overdispersion [11].

There are two main strategies for coping with fire within Mediterranean-climate floras. Resprouters survive fire and regenerate from lignotubers, epicormic buds or other structures, and reseeders are killed by fire and replaced by seedlings. Although there are different degrees and modes of resprouting, in Mediterranean-climate shrublands regeneration mode is usually considered a simple dichotomous variable [21]. Essentially, therefore, the flora is divided into two components with fundamentally different demographics: fire-resistant (resprouter) populations are relatively stable, persistent and impervious to disturbance by fire, and fire-killed (reseeder) populations are more variable and susceptible to local extinction after fire [18,21,22].

Do these ecological differences between resprouters and reseeders generate different patterns of association between phylogenetic relatedness, phenotypic similarity, and co-occurrence? Several predictions can be made:

(1) If resprouters are longer-lived with more stable populations, co-occurrence among resprouter species may be influenced by competition for space, soil moisture or nutrients, and by differentiation of species along niche axes associated with the pre-emption of these resources. Among reseeders, on the other hand, co-occurrence may be mediated by the frequency of fire, and its influence on dispersal and colonization, rather than by niche differentiation [22,23]. Under this scenario we would predict phylogenetic and phenotypic repulsion among resprouters, but not among reseeders.

(2) Alternatively, competition may be intense among reseeders, because the pressure to grow rapidly to maturity and set seed within the average fire interval is traded off against the costs of faster growth [24,25]. Hence, co-occurrence among reseeders may be controlled by niche differentiation along life-history axes associated with time to maturity, or environmental gradients such as soil fertility, which influences growth rates [22]. Under this scenario, we would predict phylogenetic and phenotypic repulsion among reseeder species but not among resprouters.

(3) Finally, there may be no difference in the mechanisms of co-occurrence between species differing in regeneration mode, if co-occurrence is determined primarily by processes that apply regardless of regeneration mode.

As a case study for testing these predictions I use the genus *Banksia*, one of Australia’s iconic plant genera and a prominent part of the flora of southwestern Australia. The genus includes 170 species that have radiated into a variety of growth forms, from prostrate ground-covers to shrubs and trees >6 m in height, and includes resprouters and reseeders. Local-scale diversity of *Banksia* is high: up to twelve species have been recorded from single 10 m x 10 m plots [26]. The analyses presented here are based on surveys of species within plots of two sizes: 20 m x 20 m (0.04 ha) and 200 m x 200 m (4 ha). I begin by quantifying patterns of co-occurrence among pairs of *Banksia* species within these plots. I then test whether co-occurrence among species pairs is associated with phylogenetic relatedness, regeneration mode, ecological similarity, and similarity in soil type preferences. I interpret co-occurrence patterns in the context of the phylogenetic signal in the different niche dimensions.

## Methods

### *Banksia* surveys

**Figure 1:**
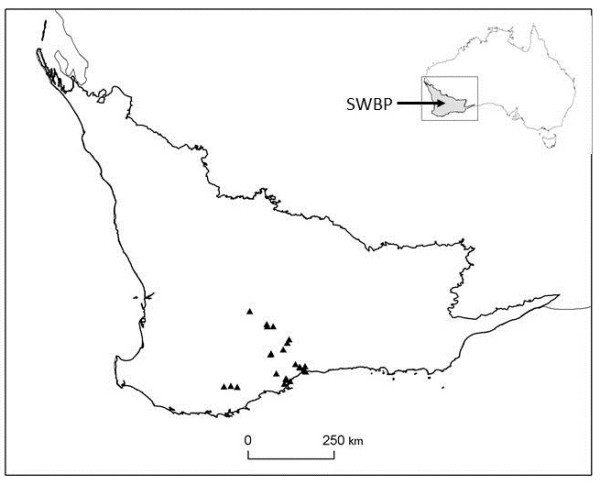
**Map of the SWBP showing the 24 study sites (triangles)**.

*Banksia* species were surveyed at 24 shrubland sites spread across an arc of the Southwest Botanical Province (SWBP) spanning approximately 250 km (Figure 1). At each site, a 200 m x 200 m (4 ha) plot was established and surveyed for the presence of *Banksia* species. Each 4 ha plot was divided into four quadrants, and one 20 m x 20 m (0.04 ha) plot was randomly placed within each quadrant. In each 0.04 ha plot, the presence and abundance (number of individual plants) of each *Banksia* species was recorded. The survey data thus comprised species presences within 24 large plots and abundances within 96 small plots. Species abundances were estimated for each 4 ha plot by taking the mean value across the four 0.04 ha plots within it. The species x sites matrices are provided in Additional file 1.

### Phylogeny reconstruction

The phylogeny used in this study is a subset pruned from a larger phylogeny of 198 *Banksia* taxa. This phylogeny was constructed from a combination of chloroplast DNA sequences previously available on GenBank (88 taxa) and newly-generated sequences (110 taxa).Most of the newly-generated sequences are for species of the former genus *Dryandra* which has recently been merged with *Banksia*[27], but has not previously been the subject of a complete phylogenetic study. The complete phylogeny of 198 taxa, with a full description of the methods of DNA extraction and sequencing, and phylogeny reconstruction, will be published elsewhere (Cardillo & Pratt, unpublished ms), but below I present a summary of the methods.

Total genomic DNA was extracted from tissue samples of both fresh material and dried herbarium specimens for 110 *Banksia* taxa (including full species, subspecies and varieties), using a Qiagen DNEasy Plant Mini Kit according to the manufacturer’s protocol. Sequencing was carried out at Macrogen, Seoul, Korea; 3656 bp of sequences across five non-coding chloroplast regions (rpl16 intron , psbA/trnH spacer, trnL intron, trnL/trnF spacer, trnT/trnL spacer) were obtained. The 110 new sequences were combined with the 88 sequences from Genbank, then aligned manually using Geneious v5.6.3 [28]. The phylogeny was estimated together with divergence times using a Bayesian analysis in BEAST 1.6.2 [29]. Calibrations were based on ages of the *Banksia* crown (42Mya, lognormal prior) and stem (62Mya, lognormal prior) estimated from fossil data [30]. To constrain the age of the root node, a normal prior with mean 77Mya was used, based on the estimated divergence time between the lineages leading to *Banksia* and *Hakea / Grevillea* in a recent genus-level phylogeny of the Proteaceae [31]. A Yule prior was used for the speciation process, and an uncorrelated lognormal model for variation in rate of molecular evolution [32]. The function modelTest() in the R library phangorn [33] was used to choose an HKY + gamma substitution model. Two separate MCMC chains were run for >40,000,000 generations, sampling trees every 10,000 generations. Sampling adequacy was diagnosed by examining effective sample sizes (ESS) using Tracer 1.5 [29]; the runs were terminated when all parameter values had ESS > 300.

**Figure 2:**
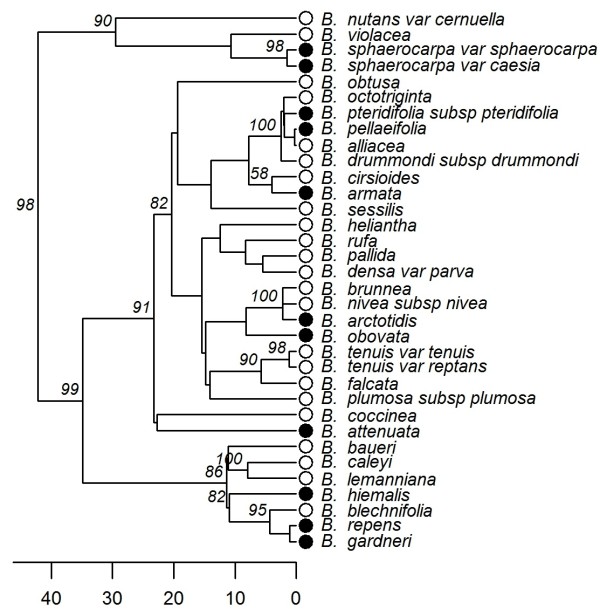
**Phylogenetic relationships among 34 of the *Banksia *species included in this study**. The phylogeny shown is from the Bayesian maximum clade credibility tree based on cpDNA sequences. Posterior support values (%) are shown for nodes where support is greater than 50%; for unlabelled nodes, support is less than 50%. Tip symbols represent regeneration mode (open circles = reseeder, solid circles = resprouter). Timescale is in millions of years before the present.

The resulting 198-taxon phylogenies include 34 of the 39 *Banksia* taxa found in the field plots; the maximum clade credibility tree, pruned to include only these 34 taxa, is shown in Figure 2. To account for phylogenetic uncertainty, most of the phylogeny-based analyses I present here were repeated on 1000 trees sampled evenly from the Bayesian posterior distribution, and I present results as summary values of distributions from this sample.

### Ecological and environmental data

I compiled a dataset of ecological traits for all *Banksia* species (see Additional file 2) using information from the standardized species entries in the Flora of Australia [34,35] and in The Dryandras [36]. Data for the following variables were recorded: (1) maximum height (species listed as prostrate were given a height of 0.2 m); (2) mean seed length; (3) fire regeneration mode (resprouter /reseeder); (4) months of the year in which flowering occurs. Height and seed size are both key indicators of major axes of variation in life history strategies of plant species [37-39], and the timing of flowering may be involved in mediating competition and structuring communities [40,41].

Many SWBP species display a high degree of edaphic specialization [42], and the fine-scale mosaic of soil types is suspected to play a role in generating and maintaining the region’s high species richness [14,43]. I therefore focused on edaphic gradients to analyse the influence of environmental filtering. In each 0.04 ha plot I collected four soil samples, which were aggregated and analysed by the Environmental Analysis Laboratory, Southern Cross University, for concentrations of calcium, potassium, nitrate, phosphorus and silicon, and for pH and organic matter content (see Additional file 3).

### Accounting for non-overlapping geographic distributions

Where species have originated allopatrically and remained geographically separated, metrics of co-occurrence of species across a set of sites could detect significant negative co-occurrences that may simply be the result of speciation history rather than species interactions or other community assembly processes. This is likely to be common in a group such as *Banksia,* in which a large proportion of species have highly restricted geographic distributions. To minimize this effect I omitted from the analyses pairs of species with no overlap in geographic distribution. Geographic distributions of species were inferred from georeferenced herbarium records obtained from the Australian Virtual Herbarium (http://chah.gov.au/avh/). I removed records with clearly erroneous location data (e.g. in the sea) or those well outside a species’ distribution limits indicated in FloraBase [44]. I then obtained an estimate of the extent of occurrence of each species by using ArcGIS [45] to draw a minimum convex polygon around the set of record locations. These are not intended to be highly resolved reconstructions of the boundaries of species’ distributions; they are simply approximations which I used only to judge whether pairs of species overlap geographically. I omitted species pairs with no overlap from further analyses of distance matrices.

### Co-occurrence of species pairs

After omitting non-overlapping species pairs, the 39 species of *Banksia* provided 419 pairwise species comparisons. For each pair, I calculated Schoener’s index of co-occurrence C*ij*, a measure of co-occurrence that takes into account the presence or absence of species at each site, as well as their proportional abundances [46]. Co-occurrence was calculated for both plot sizes (0.04 ha and 4 ha), and separately for species pairs where both species are reseeders (174 pairs), where both are resprouters (48 pairs), and where the two species differ in regeneration mode (197 pairs). The calculation of C*ij* was done using the “species.dist” function in the R library Picante [47].

### Associations between co-occurrence, phylogenetic and ecological distance

Of the 39 *Banksia* species found in the field plots, 34 were included in the phylogeny. Phylogenetic distances among these 34 species were measured as the total length of the branches connecting each pair of species. Differences in height, seed size and length of the flowering period were calculated among all of the 39 species in the field plots. To measure overlap in flowering periods for each species pair, I calculated the number of months in which both species flower, and divided this by the shorter of the lengths (in months) of the flowering periods of the two species. I then subtracted this value from one so that a larger value corresponds to greater dissimilarity in flowering period, for consistency with the other measures of ecological distance. To quantify species’ edaphic preferences, I calculated linear regressions of species’ log-transformed abundances against the values of each soil type variable, across the 96 small plots. Following Helmus *et al.* (2007) [8] I took the slopes of these regressions, whether or not they differed significantly from zero, as a measure of each species’ association with each soil type variable. The distances between species in these variables were simply the differences in the regression slopes.

All tests for associations among pairwise distance values were done using quantile regression with bootstrapped p-values. Because each species contributes to multiple species pairs, degrees of freedom are inflated and p-values based on standard significance tests are misleading. Bootstrapping avoids this problem by randomizing the dataset and testing significance by comparing p-values to the distribution of random p-values. I tested for associations between co-occurrence and phylogenetic distance, and separately for associations between co-occurrence and each ecological and edaphic distance measure. These tests were done for both 0.04 ha and 4 ha plot sizes, and for resprouters, reseeders and resprouter vs. reseeders separately. Quantile regressions were done using the “quantreg” library in R (http://cran.r-project.org/web/packages/quantreg).

### Measuring phylogenetic signal in ecological traits and edaphic preferences

Associations between co-occurrence and phylogenetic, ecological and edaphic distances can only be properly interpreted if we know the degree to which ecological traits and edaphic preferences are phylogenetically conserved or labile. To measure the strength of phylogenetic signal in height, seed size, length of flowering period and edaphic preferences, I used Î, a branch-length transformation parameter of the phylogenetic GLS model [48]. I tested the maximum-likelihood estimate of Î against hypotheses of Î = 0 (no phylogenetic signal) and Î = 1 (phylogenetic signal consistent with Brownian motion model of trait evolution). To test phylogenetic signal in regeneration strategy, I used the D statistic, which is appropriate for use with binary traits [49]. The Î and D statistics were calculated using the “lam.test.single” and “phylo.d” functions in the R library caper (http://r-forge.r-project.org/projects/caper). To measure phylogenetic signal in the overlap in species’ flowering periods, I used quantile regression to test for significant associations with phylogenetic distance across species pairs.

I then looked for evidence that recently-separated lineages have diverged rapidly with respect to each niche axis. To do this, I plotted the ages of nodes in the *Banksia* phylogeny against standardized contrasts in the ecological and soil-preference traits at each node. Typically, contrasts are standardized to a common variance by dividing their absolute value by the square root of the variance expected under a Brownian motion model of trait evolution, which corresponds to the sum of the branch lengths at each node [50]. However, particularly for labile traits, this can result in overstandardization, where contrasts become smaller at deeper nodes because they are being divided by a larger number [51]. To minimize this effect, Garland *et al.* (1992) recommended transforming the branch lengths of the tree prior to standardizing contrasts. Here I transform branch lengths by raising them to a power Îº, then optimizing the value of Îº to minimize the correlation between absolute standardized contrasts and their standard deviations [51]. The resulting plots give a visual guide to the patterns of ecological divergence between lineages in each variable. Significant positive outliers on these plots can be interpreted as lineages that have diverged more than expected from their age of separation. Because the branch-length optimization renders slope estimates non-comparable, I applied this test only to the maximum clade credibility tree from the Bayesian analysis, not to the sample of 1000 posterior trees. Contrasts were calculated using the “crunch” function in the R library caper (http://cran.r-project.org/web/packages/caper), and functions written by A. Purvis (unpublished).

## Results

Across all geographically-overlapping species pairs, there is a positive association between phylogenetic distance and the co-occurrence metric C*ij* (Table 1). This indicates a general pattern of phylogenetic repulsion, with co-occurrence less likely among closely-related species. When resprouters and reseeders are tested separately, phylogenetic repulsion is evident among pairs of reseeder species, but there is no significant association among the resprouter species (Table 1). Among species pairs that differ in regeneration mode, the positive association between phylogenetic distance and C*ij* remains significant.

**Table 1:** Slopes of quantile regressions for phylogenetic and ecological distance

**Data subset**	**Plot size (ha)**	***d.f.***	**Phylogenetic distance**	**Height**	**Seed size**	**Flowering period length**	**Flowering period overlap**
all species pairs	0.04	417	0.003(0.002, 0.004)***	0.03	0.001	0.01	−0.02
	4	417	0.004(0.002, 0.006)**	0.06**	0.002	0.02	−0.07
resprouters only	0.04	46	−0.003(−0.006, 0.003)	−0.01	−0.003	−0.03	0.03
	4	46	−0.006(−0.01, 0.003)	−0.01	−0.003	−0.03	0.05
reseeders only	0.04	172	0.003(0.002, 0.005)*	0.08***	0.0003	0.03	−0.03
	4	172	0.004(0.003, 0.007)†	0.08***	0.0008	0.03	−0.04
resprouters vs. reseeders	0.04	195	0.002(0.001, 0.003)*	−0.005	0.003	0.009	−0.05
	4	195	0.003(0.002, 0.005)*	−0.007	0.004	0.008	−0.09

Response variable is co-occurrence among species pairs. Slope values for phylogenetic distance are medians (with 2.5^th^ and 97.5^th^ percentiles) from 1000 trees sampled from the Bayesian posterior distribution.

***p ≤ 0.001; **p ≤ 0.01; *p ≤ 0.05; †*p* ≤ 0.1.

There are positive associations between C*ij* and plant height, for all species pairs and for reseeders only, but no association for resprouters only, or for reseeders vs. resprouters (Table 1). This indicates phenotypic repulsion among reseeders, such that pairs of species of similar height are less likely to co-occur. There are no significant associations between co-occurrence and seed size, length of the flowering period or degree of overlap in the flowering period (Table 1).

Among the soil type variables, nitrate and pH show negative associations with C*ij* across all species pairs (Table 2). These associations are not significant among resprouters only or reseeders only*,* but are significant among species pairs that differ in regeneration mode. This indicates a pattern of environmental attraction among species that differ in regeneration mode: species are more likely to co-occur if they are similar in their response to soil nitrate concentration or pH.

**Table 2:** Slopes of quantile regressions for soil type preferences

**Data subset**	**Plot size (ha)**	***d.f.***	**Calcium**	**Potassium**	**Phosphorus**	**Nitrate**	**pH**	**Organic matter**	**Silicon**
all species pairs	0.04	417	10.86	−30.93	−0.53	−13.25*	−1.49***	−0.93	−24.37
	4	417	108.1	−68.25	−1.08	−16.56*	−2.25***	0.66	−81.15
resprouters only	0.04	46	134.8	−270.41*	−0.56	−18.45	−1.75	−4.35	−284.31
	4	46	228.11	−270.41	−0.97	−18.45	−1.75	−4.46	−361.93
reseeders only	0.04	172	110.19	−18.64	−0.7	2.72	−1.84	0.83	17.91
	4	172	62.91	−43.89	−1.3	−5.47	−2.49	1.04	−77.57
resprouters vs. reseeders	0.04	195	−83.04	−47.1	−0.49	−17.63*	−1.41*	−0.95	−36.73
	4	195	35.39	−94.18	−0.79	−30.97**	−2.5*	0.56	−88.83

Response variable is co-occurrence among species pairs. ***p ≤ 0.001; **p ≤ 0.01; *p ≤ 0.05; †*p* ≤#8201;0.1.

Tests for phylogenetic signal in ecological traits (Table 3) show that plant height and seed size are phylogenetically conserved, with median maximum likelihood estimates of λ not significantly different from a Brownian model (λ =1), while the length of the flowering period is labile (median λ ≈ 0). Regeneration mode is labile, with median values of D rejecting a Brownian model (D = 0). The timing of the flowering period, as measured by quantile regression of flowering period overlap x phylogenetic distance across species pairs, is labile (slope ≈ 0 and *p* ≈ 1 for all 1000 trees). Species preferences in all edaphic variables, with the exception of organic matter, are phylogenetically labile (Table 3), with median maximum likelihood estimates of λ not significantly different from λ = 0, and significantly different from λ = 1. Preference for organic matter content, on the other hand, appears to be conserved (Table 3).

**Table 3:** Phylogenetic signal in ecological traits and edaphic preferences

	**Test statistic**	**p(labile)**	**p(Brownian)**
Ecological traits			
height	λ = 0.98(0.94,1)	0(0,0)	0.25(0,1)
seed size	λ = 0.96(0.84,1)	0(0,0)	0.07(0,1)
regeneration mode	D = 0.9(0.57,1.16)	0.35(0.09, 0.68)	0.02(0.001, 0.11)
flowering period length	λ = 0.0001(0.0001,1)	1,(0.009,1)	0.004(0,1)
Soil preferences			
calcium	λ = 0.0001(0.0001,0.98)	1,(0.15,1)	0.008(0,1)
potassium	λ = 0(0,0)	1,(1,1)	0(0,0.01)
phosphorus	λ = 0.0001(0.0001,09)	1,(0.06,1)	0.0001(0,0.16)
nitrate	λ = 0(0,0)	1,(1,1)	0(0,0.004)
pH	λ = 0(0,0)	1,(1,1)	0(0,0)
organic matter	λ = 0.99(0.0001,1)	0.001,(0,1)	1(0.002,1)
silicon	λ = 0(0,0)	1,(1,1)	0(0,0.03)

For regeneration mode the test statistic is D, for all other variables the test statistic is λ. Values shown are medians (with 2.5^th^ and 97.5^th^ percentiles) from 1000 trees sampled from the Bayesian posterior distribution.

**Figure 3:**
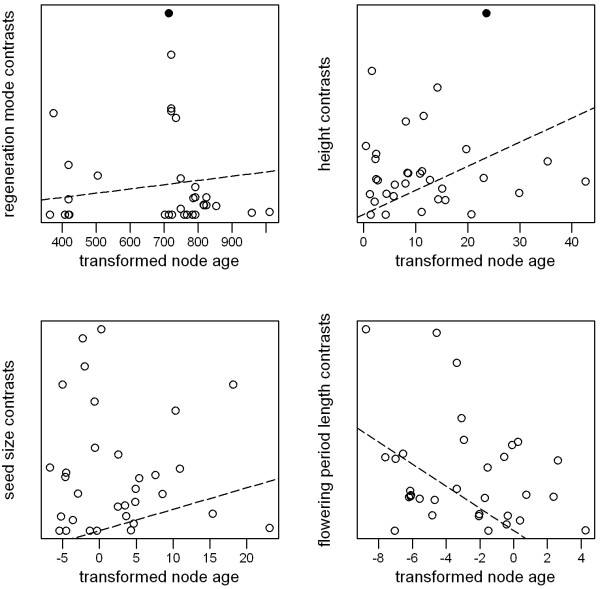
**Plots of node ages against standardized contrasts in ecological traits.** Node ages are based on transformed branch lengths; see text for details. Points are nodes in the *Banksia* phylogeny; dashed lines are least-squares regressions through the origin; solid circles are significant outliers from the regression line (absolute studentized residual > 3), indicating nodes with a greater divergence in a given trait than expected from their age.

**Figure 4:**
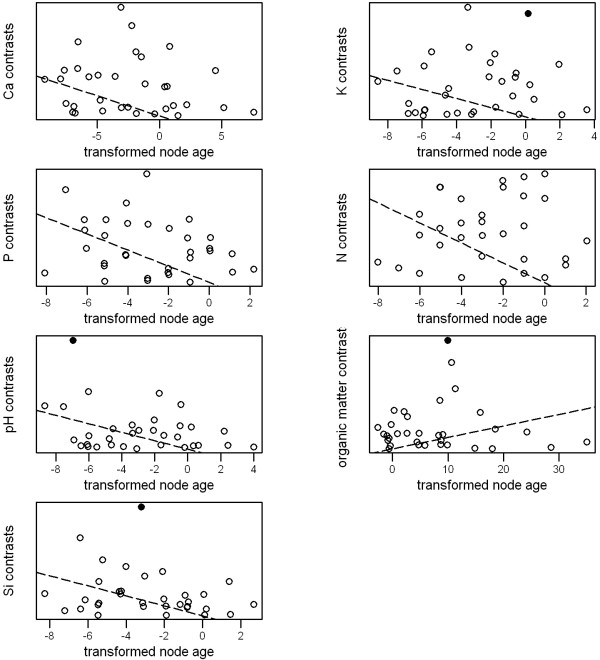
**Plots of node ages against standardized contrasts in species’ edaphic preferences.** Node ages are based on transformed branch lengths; see text for details. Points are nodes in the *Banksia* phylogeny; dashed lines are least-squares regressions through the origin; solid circles are significant outliers from the regression line (absolute studentized residual > 3), indicating nodes with a greater divergence in a given edaphic preference than expected from their age.

Plots of node ages against standardized contrasts provide little evidence that closely-related lineages have diverged rapidly with respect to ecological traits or edaphic preferences (Figures 3 and 4). Although many of the slopes of these plots are negative, there are few significant outliers, and few nodes for which divergences are greater than expected from their ages.

## Discussion

The degree to which the high species richness of Mediterranean-climate shrublands is maintained by competition-driven differentiation of functional traits [11,20], or by lottery recruitment and disturbance-mediated coexistence [14,52], is not fully clear. These two models make different predictions about the associations between species co-occurrence, phylogenetic relatedness, and phenotypic similarity. I have shown that for *Banksia* in southwestern Australia, these associations vary between fire-killed and fire-resistant species, suggesting that the ecological processes that govern co-occurrence and community structure do not necessarily apply uniformly to all species within the same communities.

The three scenarios entertained in the Introduction were that (1) patterns for resprouters but not reseeders are consistent with niche differentiation; (2) patterns for reseeders but not resprouters are consistent with niche differentiation; (3) there are no differences in patterns between resprouters and reseeders. The results provide greatest support for scenario 2, with reseeders, but not resprouters, showing patterns of phylogenetic and phenotypic repulsion consistent with niche differentiation. Of the traits included in this study, maximum adult height appears to play a role in driving the phylogenetic signal of co-occurrences among reseeders, as it shows a pattern of phenotypic repulsion and is strongly phylogenetically conserved. In contrast, the results provide no evidence that seed size, or the length or degree of overlap in flowering period, are associated with patterns of co-occurrence. The adult height attained by a plant species represents a tradeoff between the benefits of greater height, such as greater light interception and seed production, and the costs, such as greater proportional investment in non-reproductive support tissue [24,25]. Height is therefore a key dimension of ecological variation in plants [38] and a potential axis for niche differentiation where competition between species exists [53,54]. The plots of contrasts in height against node ages show no evidence for rapid divergence among closely-related lineages. Height therefore appears to influence the co-occurrence patterns of reseeding *Banksia* species by ecological sorting (i.e. competitive exclusion) rather than through microevolutionary divergence.

Why should there be evidence of niche differentiation along the axis of height for reseeders, but not for resprouters? There is likely to be selective pressure on reseeder species to grow rapidly and reach full seed production within the average fire recurrence period, favouring a lower adult height [55]. This is traded off against the costs of faster growth and lower adult height, including greater soil nutrient requirements, lower total seed production and reduced seed dispersal capacity [22,25,53]. On the other hand, it is less easy to explain why these selective pressures should be absent in resprouters. It has been observed that some resprouting *Banksia* species have very low rates of recruitment from seed, and most plants present after a fire are adults that have resprouted, rather than new recruits [23,55]. Furthermore, adult height does not reflect longevity in resprouters as it does in reseeders. Hence, the costs and benefits of rapid growth and lower height may be less critical for resprouters compared to reseeders. Of course, it is also possible that because of the smaller number of pairs of resprouter species (48 compared to 174 reseeder pairs), there was simply less power to detect patterns of phylogenetic and phenotypic repulsion. Another possibility is that there may be other niche dimensions not included in my study that influence co-occurrence patterns among resprouters.

The patterns of phenotypic repulsion based on height are set against a background of environmental filtering, whereby species with similar edaphic preferences (with respect to soil nitrate concentration and pH) are more likely to co-occur. This is consistent with the high degree of specialization to soil types that seems to characterize old, nutrient-impoverished landscapes in southwest Australia and elsewhere [56]. However, it is not obvious why *Banksia* co-occurrences should be associated with soil nitrate rather than with phosphorus, which is more likely to be the limiting nutrient in the highly weathered soils of southwestern Australia [56,57]. One possibility is that the measures of phosphorus concentration used here were for total phosphorus rather than plant-available forms. Unlike height, species’ edaphic preferences are highly labile, so this pattern is essentially independent of relatedness, and thus leaves no signature in the phylogenetic patterns of co-occurrence. The lability of edaphic preferences is consistent with the hypothesis that local-scale specialization to soil types happens rapidly, potentially contributing to rapid speciation rates in Mediterranean-climate shrublands [14,15,58].

My approach to analyzing community phylogenetic patterns based on pairwise co-occurrences is similar to that employed for schoenoid sedges in another Mediterranean-climate shrubland, the Cape Floristic Region, which also found evidence for phylogenetic repulsion [11]. This approach differs from a recent study by Merwin *et al.*[12] that analyzed phylogenetic structure in *Banksia* communities using whole-assemblage metrics. In contrast to my finding of phylogenetic repulsion among species pairs, Merwin *et al.* found that many communities were phylogenetically clustered, indicating that closely-related species are more likely to co-occur. This contrast can most likely be explained as an issue of spatial scale. Their plots spanned a far greater geographical area than mine, and they interpret phylogenetic clustering as the signal of speciation and limited dispersal abilities generating many closely-related but narrowly-distributed species within a given region. In contrast, not only were my plots spread over a smaller area, but I explicitly attempted to minimize the signal of speciation history by omitting non-overlapping pairs of species from the analysis. In this way, my analyses of co-occurrence patterns were more likely to have detected local-scale ecological patterns rather than broad biogeographic effects.

Similarly, my finding of phylogenetic repulsion appears at odds with another recent study that showed that fire regeneration mode can drive phylogenetic clustering through shared adaptation of close relatives to fire-prone environments [13]. Again, the difference can be explained by a difference in scale: the study by Verdu & Pausas [13] examined patterns across two major habitat types, one of which was fire-prone and the other not. At these scales, environmental filtering is likely to predominate over competition; furthermore, fire response in the flora they examined was phylogenetically conserved. This contrasts with *Banksia* in which regeneration mode is highly labile and its primary influence on community structure appears to be by mediating competitive interactions among species.

## Conclusions

Phylogenies are now an integral part of community ecology, and the analysis of community structure and co-occurrence in the context of phylogenetic relatedness has contributed a great deal to our understanding of the ecological processes that govern species distributions and community assembly. However, community phylogenetic studies still commonly assume an overarching ecological structuring process for all species in a community, at a given spatial and phylogenetic scale. The different patterns for fire-killed and fire-resistant *Banksia* species suggest that this assumption may be simplistic, and that species with different ecological and demographic attributes, and subject to different selective pressures, can show different patterns of phylogenetic and phenotypic structure. Therefore, whole-community metrics of phylogenetic structure such as NRI and NTI may not be the most powerful way of understanding community assembly processes, and analyses of pairwise co-occurrence patterns may be more informative.

## Competing interests

The author declares that he has no competing interests.

## Supplementary Material

Additional file 1 Species x sites matrices. Click here for file

Additional file 2 Species ecological trait data. Click here for file

Additional file 3 Soil data. Click here for file
